# Mine Tailings as Supplementary Cementitious Materials: Properties, Performance, Mechanochemical Activation, and Challenges

**DOI:** 10.3390/ma19204276

**Published:** 2026-10-10

**Authors:** Kyriaki A. Papadopoulou, Magdalena Rajczakowska, Lena Alakangas, Andrzej Cwirzen

**Affiliations:** Department of Civil Engineering, Environmental and Natural Resources, Luleå University of Technology, 97187 Luleå, Sweden; magdalena.rajczakowska@ltu.se (M.R.); lena.alakangas@ltu.se (L.A.); andrzej.cwirzen@ltu.se (A.C.)

**Keywords:** mine tailings, supplementary cementitious materials (SCMs), mechanochemical activation (MCA), high-energy grinding

## Abstract

The environmental impacts associated with Portland-cement production have driven decades of research into sustainable supplementary cementitious materials (SCMs). An emerging alternative, mine tailings, has been gaining attention lately due to its large availability, environmental burden, and potential for resource recovery. The paper reviews the use of mine tailings in concrete and their potential as an alternative SCM source. Fundamental properties, such as chemical composition, mineralogy, crystalline structure, fineness, and amorphous content, are reviewed for their influence on the behavior of mine tailings in cement-based systems. With low reactivity and variable compositions posing the main restrictions, mechanochemical activation (MCA) is assessed as an effective strategy to enhance their performance. By reducing the particle size, increasing the specific surface area, and inducing structural disorder, mechanochemical treatment can improve the pozzolanic or filler activity of mine tailings and thereby increase their suitability for blended cement applications. Overall, this structured narrative review highlights mechanochemically activated mine tailings as a potential pathway toward low-carbon concrete and circular material management, all while highlighting the need for further research that will provide additional insights regarding the correlations between mine tailings’ properties, milling conditions, and reactivity, as well as the long-term durability of concrete incorporating them.

## 1. Introduction

According to the United Nations, the current world population is more than three times larger than it was 100 years ago. It is expected to reach around 8.5 billion in 2030, and 9.7 billion in 2050, of which 7 billion are expected to live in urban habitats [1]. The ever-growing population, globalization, urbanization, and industry growth correspond to a simultaneous increase in the need for clean water, clean air, safe transportation, and natural energy sources for public, industrial, and residential buildings [2].

Concrete is already the second-most widely used material in the world after water and the most widely used construction material compared to steel, wood, plastic, and aluminum [3,4]. Concrete has become and still remains the primary construction material because it combines broad geographic availability of components, comparatively low unit cost in many markets, adaptable fresh-state behavior, and adjustable hardened performance [3,5,6]. Its components can generally be supplied through established regional networks, while fresh concrete can be mixed, transported, pumped, poured, compacted, or precast into a wide range of geometries [7]. Water-to-binder (w/b) ratios, aggregate grading, SCMs, chemical admixtures, reinforcement or prestressing can be adjusted to fulfill different workability, setting time, strength, durability, and service-life requirements [5]. Concrete’s continuous use is supported by existing and established supply chains, design codes, standards, specialized equipment, experienced workforces, and public trust, all of which reduce implementation risk and create practical barriers to rapid substitution by alternative approaches [6].

The annual use of concrete is estimated to be around 11 billion metric tons [4,5], which translates into more than 4 billion tons of cement being produced annually and accounts for approximately 8% of global anthropogenic CO_2_ emissions, yielding an annual production of roughly 2 tons of concrete for every person on the planet [7]. The construction sector in Europe alone accounts for 36% of the overall CO_2_ emissions and for 40% of the overall energy consumption [8]. As a composite material, concrete consumes large amounts of natural resources (water, rock, sand, gravel, stone, etc.), directly or indirectly sourced from the Earth’s crust, in addition to carbon emissions and energy consumption. A major percentage of the environmental impact of concrete derives from cement manufacturing, with the entire clinkerization process consuming ~3.5 GJ/ton [9]. As of 2019, the demand for aggregates alone increased by ~5.2%, with China accounting for nearly half of this demand [10]. Combined with the above-mentioned population growth projections, civil infrastructure is also expected to increase to cover the needs of future urban societies. According to the Organization for Economic Co-operation and Development (OECD), a fast-moving rise in production and construction material usage is expected in the next few decades. It is becoming increasingly evident that the industry needs to shift towards more viable, sustainable, and environmentally friendly technologies [11].

Despite their heavy carbon footprint, shifting away from conventional processes is difficult, as concrete and its related cement-based materials provide an easy and affordable construction practice [7]. The European Cement Association and the International Energy Agency have recently emphasized the urgency of exploring alternatives to conventional raw material resources to prevent the eventual depletion of the Earth’s crust, as well as alternative binder options [10,12,13,14].

Mine tailings are generated in large volumes, have already undergone comminution during mineral processing, and commonly contain silicate or aluminosilicate phases, making them a potential source of supplementary cementitious materials (SCMs) worthy of investigation [7,10,15,16,17]. Their incorporation into cement-based materials could reduce both the demand for clinker and raw materials, as well as the volume of residues that require long-term storage [6,7,15,18]. However, their suitability as SCMs is not intrinsic and depends on their mineralogical composition, amorphous content, particle-size distribution, chemical reactivity, and potentially hazardous constituents, as well as the additional processing required before use [7,17,19,20,21,22,23,24].

Existing research is scattered across a variety of applications (e.g., concrete, alkali-activated binders, ceramics, aggregates, backfill, etc.) [7,10,15,22,25] and activation methods (i.e., thermal, chemical, mechanical, and hybrid routes) [22]. Studies on emerging SCMs emphasize direct reactivity testing and the need to separate chemical contributions from filler effects [19,20,21,26,27,28,29,30].

This structured narrative review aims to identify and organize the available studies on the use of mine tailings as SCMs, to evaluate the potential of this approach, and to critically assess the conclusions that can be drawn from the existing evidence. Additionally, it examines the potential of mechanochemical activation as a treatment for the revalorization of tailings before being incorporated into cement-based applications. Emphasis is deliberately given on MCA because, while thermal and chemical activation has been more broadly established in the literature, MCA of mine tailings remains an emerging, predominantly laboratory-scale approach in which correlations between mineralogy, milling conditions, energy input, structural disorder or amorphization, chemical reactivity, and industrial scalability remain insufficiently resolved [23,31,32,33]. Strengths, limitations, and unresolved questions are determined to establish research priorities that will uncover the criteria under which tailing-derived SCMs can provide technically credible and environmentally beneficial partial replacements for Portland cement.

## 2. Review Methodology

This study was designed as a structured narrative review addressing three questions:

(i) Which properties determine the suitability of mine tailings as supplementary cementitious materials?
(ii) How does mechanochemical activation modify tailings’ structure, dissolution, and reactivity?
(iii) What evidence is there that supports performance, environmental-safety, and industrial-feasibility claims?

A narrative synthesis was selected because the available studies differ substantially in tailing source, mineralogy, activation treatment, mixture design, replacement level, curing regime, and outcome definition, precluding a meaningful quantitative meta-analysis.

Scopus, Web of Science, Springer Materials, ResearchGate, and ScienceDirect were searched. Google Scholar was used for supplementary searching, with backward reference and forward citation tracking. Standards, policy documents, and industry roadmaps were identified through the official websites or catalogs of the relevant European standards bodies, the European Commission, the International Energy Agency, the Global Cement and Concrete Association, and the United Nations.

Searches combined terms for the material source (tailing*, mine tailing*, mining tailing*, mine waste*, mining waste*, and mineral waste*, as well as mineral-specific variants, such as iron ore, copper, gold, phosphate, tungsten, graphite, lead–zinc, bauxite, kaolin, and coal mining waste) with terms for cement and concrete (supplementary cementitious material*, SCM, pozzolan*, cement replacement, blended cement, cement, concrete, mortar, and binder). Searches on activation treatments added mechanochemical activation, mechanical activation, high-energy grinding, milling, ball mill, planetary mill, disc mill, stirred mill, amorphization, and structural disorder. Searches for performance evidence terms related to hydration, dissolution, calorimetry, reactivity, heat of hydration, workability, strength, durability, carbonation, chloride, sulfate, freeze–thaw resistance, permeability, leaching, heavy metal immobilization, acid mine drainage, life-cycle assessment, energy, scale-up, and cost.

Primary studies were eligible when they examined mine tailings or ore-processing residues in cement paste, mortar, concrete, or blended cement, or when they evaluated activation and reported the relevant characterization, structural change, dissolution, reactivity, hydration, mechanical performance, durability, leaching, energy, scale-up, or economic outcomes. Reviews, standards, books, and institutional reports were included when required for definitions, test requirements, mechanisms, environmental context, or policy background. Studies of alkali-activated binders, aggregates, bricks, ceramics, and backfill were retained only as contextual evidence and were not treated as direct proof that tailings constitute an SCM. Fundamental mechanochemical studies on relevant clays or minerals were included only when they addressed mechanisms insufficiently resolved in the tailing-specific literature.

Records were excluded when they did not directly investigate tailings or an explicitly relevant mechanistic analog, reported no pertinent characterization or cement-related outcome, or addressed only recovery or disposal without relevance to the review questions. Only English-language records were included.

For the included sources, data were documented (when available) on tailing origin, chemical and mineralogical composition, particle size, specific surface area, potentially hazardous constituents, intended function in the cementitious system, activation method, processing parameters, evidence of structural change and chemical reactivity, cement-replacement level, curing conditions, hydration, mechanical, and durability performance, and leaching or environmental-safety results.

All evidence was synthesized by the material function (e.g., aggregate, filler, reactive SCM, or alkali-activated precursor). Materials were described as reactive SCMs only when structural or compositional evidence was accompanied by direct reactivity measurements, such as dissolution, calorimetry, calcium-hydroxide consumption, phase evolution, or hydrate formation. Environmental and economic advantages were treated as unconfirmed unless supported by comparative life-cycle or techno-economic assessment with explicit system boundaries. Owing to the heterogeneity of materials, processing conditions, mixtures, and outcomes, the results were synthesized narratively and were not statistically analyzed.

Between January 2025 and June 2026, the literature databases and platforms listed above were revisited periodically during iterative searches and were searched without a publication-year limit. Papers with no evident relevance to the review questions upon inspection of their titles and abstracts were excluded. The remaining papers were assessed individually against the eligibility and contextual-evidence criteria described above. In interpreting the included studies, we considered the reported tailing characterizations, processing and mixture conditions, and their relevant comparisons, as well as whether direct reactivity measurements supported claims of chemical contributions, where such details were available. This was a descriptive assessment of the evidence presented and not a standardized risk-of-bias appraisal. The material-function categories and separation of direct SCM studies from contextual and mechanistic sources provided the organizing structure for the qualitative synthesis.

The above-described methodology imposes certain limitations to the current review regarding its search and selection record. Explicit search and screening logs; exact dates of individual search runs; precise records of identified, screened, or excluded studies; and counts by exclusion reason were not recorded. Therefore, the review does not claim exhaustive retrieval. Additionally, no publication-frequency analysis was performed, although a qualitative assessment of the list of studies included indicates there were very few records before the 2010s with an apparent increase thereafter. Likewise, the descriptive appraisal above should not be read as a formal risk-of-bias evaluation of the included studies. Lastly, the conclusions presented at the end of this review are a critical interpretation of an extensive, though not exhaustive, selection of evidence.

## 3. Supplementary Cementitious Materials

One of the principal strategies that has been implemented and practiced over the past few decades for the decarbonization of the cement sector has been the partial substitution of Portland cement by supplementary cementitious materials (SCMs) [6,26,34]. The main constraint of this solution is the availability of befitting SCMs and their local variability, that do not compromise the targeted performance [19,20]. For a material to be considered as a potential SCM, it must fulfill a set of requirements related to physical and chemical properties, reactivity, and mineralogy. SCMs include pozzolanic, latent-hydraulic, and mixed-reactivity materials. Their reactivity depends on the type and abundance of reactive phases, chemical composition, structural disorder or amorphous content, fineness, and the chemistry of the cementitious system [20,21,27,28]. SCMs should also have sufficient available volumes and limited negative effects on workability, hydration, strength development, durability, and environmental compatibility [28,29]. Where an SCM is covered by a national or regional product standard, the relevant compositional and performance requirements apply. Emerging SCMs that are not yet covered by prescriptive standards require a performance- and risk-based qualification [19,26]. Partially replacing Portland cement with SCMs does not directly reduce clinker carbon emissions, but it does reduce the footprint of the produced concrete [6].

SCMs can influence cement hydration through both physical and chemical mechanisms. Fine particles may improve packing, provide nucleation surfaces, alter water demand, or change the effective water-to-binder ratio, even when they remain chemically inert. Chemical contribution requires the dissolution and reaction of the material in the cementitious environment, leading to calcium-hydroxide consumption or the formation of additional hydrates [28,29]. Cement replacement by mass or retention of compressive strength is therefore not sufficient evidence of pozzolanicity.

Typical SCMs have generally derived from industrial by-products generated by coal-fired power plants and steel and ferrosilicon industries, with the most used being fly ash, ground granulated blast-furnace slag, and silica fume. The use of such side streams as SCMs has been thoroughly and extensively researched in the past few decades, turning them to conventional and standardized SCMs while giving rise to new research directions even today. Some indicative recent examples of such studies include Anjos et al. [35], who examined carbonation and chloride resistance of self-compacting concretes with fly ash; Kim et al. [36], who evaluated ferronickel slag as a partial cement replacement in concrete intended for precast applications; and Liang and Wang [37], who investigated limestone mine dust filter residue blended with fly ash in cement mortar. The availability of these SCMs is intrinsically dependent on the waste streams of industries that are currently under substantial transformation or a gradual phase-out as part of decarbonization strategies. Additionally, SCMs are not as abundant as natural raw materials, and thus are not readily available in sufficient quantities across geographical regions. In areas with limited local supply, an increase in CO_2_ emissions may arise due to higher clinker or cement consumption or from long-distance transportation of adequate SCM quantities [7,14].

This emerging and upcoming shortfall in conventional SCMs has driven interest in exploring alternative sources for SCMs—primarily waste streams and, in particular, high-volume ones. Over the last three decades, research in the field of SCMs has been growing exponentially, accumulating an extended amount of scientific knowledge which, in combination with the practical experience from the industry, has driven cement and concrete standards and specifications to start including new types of SCMs, new combinations, and even higher clinker substitution rates. The scope remains unwavering, with an additional reduction in cement-related carbon emissions.

The suitability of an emerging SCM should be assessed in stages. Bulk oxide composition provides useful screening information, but it does not show whether silicon, aluminum, or calcium occurs in a reactive phase or in stable minerals, such as quartz and feldspar. Chemical analysis should therefore be combined with quantitative mineralogy, amorphous content, particle-size distribution, specific surface area, morphology, loss on ignition, moisture, and potentially deleterious constituents [19,20,21,30]. Direct reactivity may then be evaluated through heat release and calcium-hydroxide consumption in an R^3^-type test, dissolution measurements, calorimetry, thermogravimetry, quantitative X-ray diffraction, or evidence of newly formed hydrates [20,23,30]. Final assessment should use matched cement-paste, mortar, or concrete controls with clearly reported replacement levels, water-to-binder ratios, curing conditions, and test ages.

**Figure 1:**
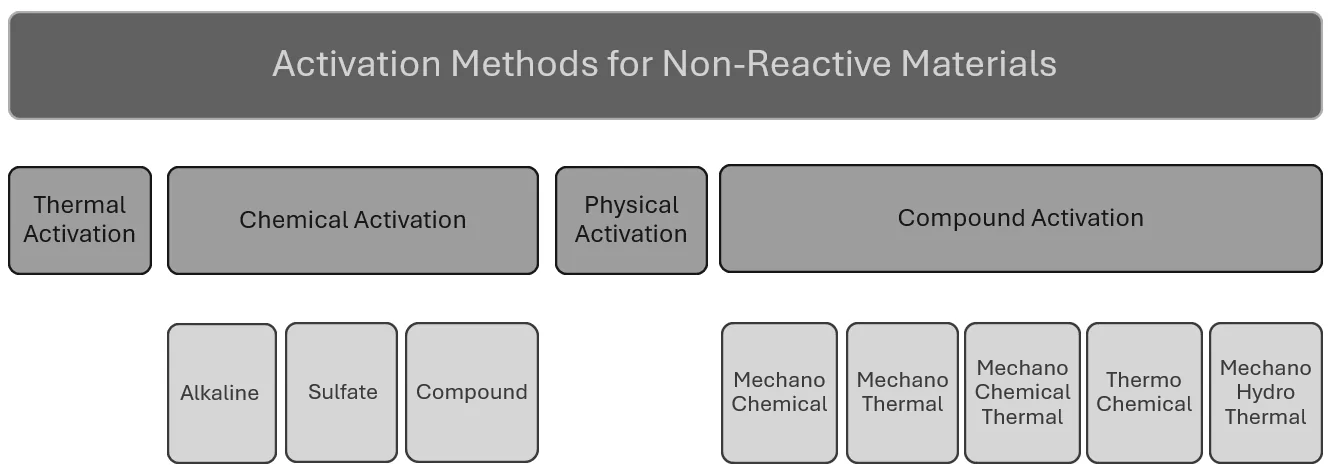
Potential activation methods for non-reactive materials (adapted from [22]).

Even though the concept of utilizing waste and by-products as SCMs is not completely new, the alternative sources that are being explored present one common challenge. Most of these waste materials are not reactive, meaning they require some level of beneficiation processing to enhance their performance and suitability as SCMs [26,30]. Systematic and targeted examination of different treatment methods that can enhance performance within cement and concrete mixes in the long term is fundamental. The available activation methods so far are thermal, chemical, mechanical, and hybridized [22]. An overview of the available activation methods is presented schematically in Figure 1.

Activation of a non-reactive or weakly reactive mineral material should be selected according to its phase assemblage and the reaction pathway required in the cementitious system. Thermal activation can dehydroxylate or transform suitable minerals and generate structural disorder; chemical activation can modify dissolution and precipitation conditions; and mechanical or mechanochemical treatment can reduce particle sizes, expose fresh surfaces, create lattice defects, and, in some materials, increase the disordered or amorphous fraction [22,30]. Hybrid treatments may combine these effects, but no activation route is universally effective. Bulk oxide composition or increased fineness alone is therefore insufficient to predict whether treatment will produce a chemically reactive SCM [20,21,30].

Activation studies should distinguish physical effects from chemical reactions. The treated material should be compared with both the untreated material and an appropriate reference under matched mixture and curing conditions. Evidence of improved packing or compressive strength should be supplemented by direct measurements of dissolution, heat release, calcium-hydroxide consumption, phase evolution, or hydrate formation where SCM reactivity is claimed [20,28,29,30]. Any performance gain should also be assessed against processing energy, durability, leaching, and practical scalability rather than treatment efficacy alone [19,22].

## 4. Mining Waste

The mining industry not only accounts for 6.9% of the global GDP but also for the generation of 7 billion tons of mine tailings yearly worldwide, including the environmental destruction caused by abandoned mines (around 10,000 abandoned mines in Canada, 50,000 in Australia, and 6000 in South Africa, as well as 9500 coal mines in China) [15]. Usually, mining activities are linked to the financial and societal development of a region. More than 17,500 companies, employing more than 500,000 people, are active in Europe alone. The Western United States is heavily developed and relies on its mining industry [38]. The already leading role of the mining industry is further highlighted by the global transition toward cleaner energy, which heavily relies on a broad range of metals and minerals, primarily sourced from ores. Consequently, the anticipated growth of the mining sector is expected to demand more land for mining operations and thus lead to a significant increase in waste production [16,39,40,41,42].

**Figure 2:**
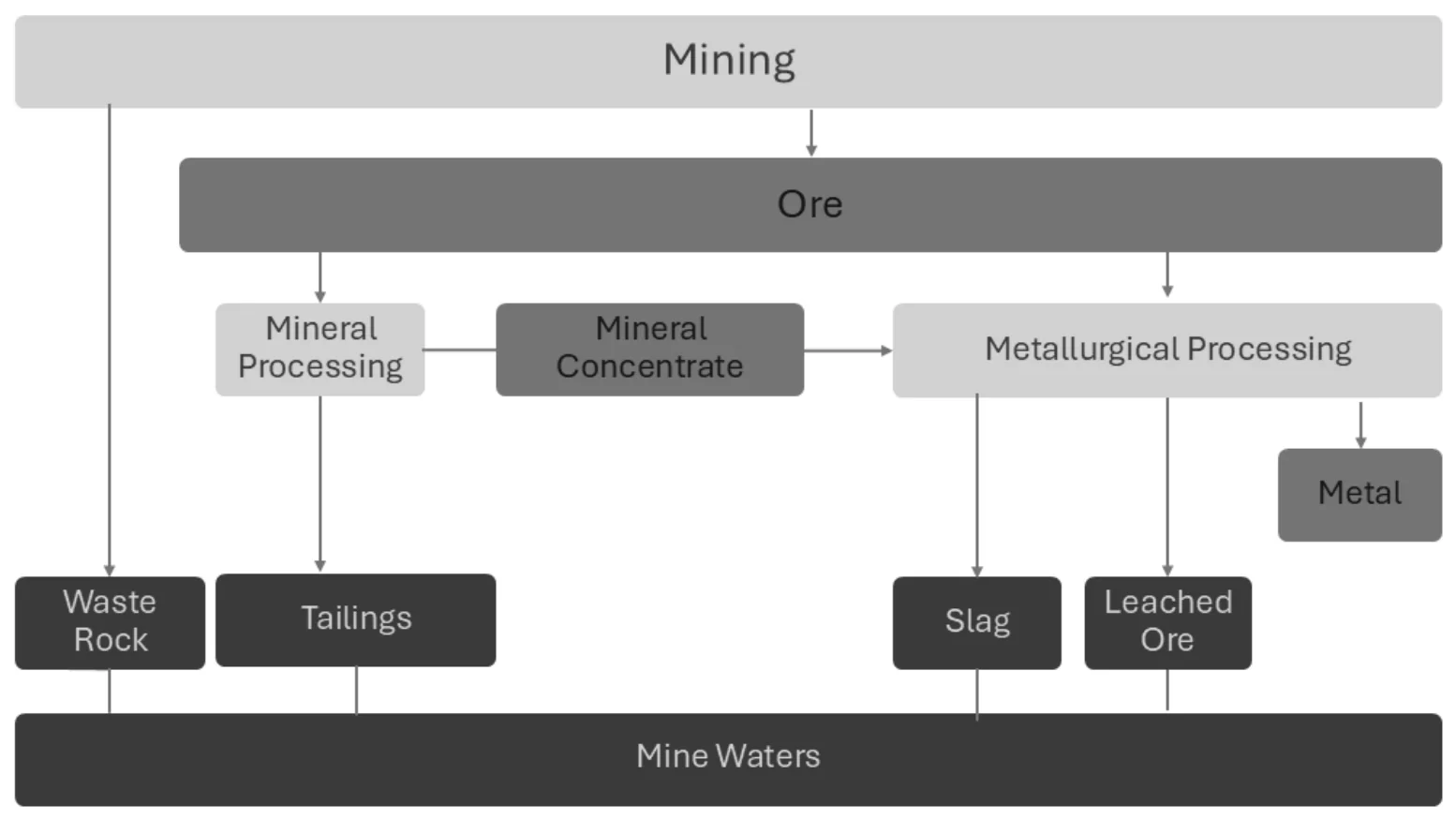
Mining waste types and their process origins (adapted from [40]).

Along with the depletion of natural resources, the large volumes of waste constantly generated by industrial and urban activities are the cause of economic and environmental issues. The mining industry is one of the major contributors towards this end, producing vast amounts of solid waste in several forms due to mining, mineral processing, and metallurgical extraction [4,43,44]. In 2002, the Mining, Minerals, and Sustainable Development Project reported approximately 3500 active mineral waste facilities around the world, mostly tailing dams and waste rock dumps [45]. Solid waste from mining operations includes rock fragments (the non-marketable part of the extracted rock), concentrator tailings resulting from the ore processing phase (consisting of gangue minerals, water, chemical additives, contaminated water treatment sludge), and dead soil after excavation [43]. The various types of mine waste and their process origins can be seen in Figure 2.

The Roadmap for Resource Efficiency by the European Commission stated that waste should be managed as a resource by 2020 [46]. As the mining sector is still trying to implement the principles of the circular economy, innovative concepts are being explored for managing, recycling, reusing, and upcycling waste. Mine waste has been recycled for several applications, which include, among others, the removal of metals from mine water and the capture of CO_2_ [47,48]. Moreover, these wastes are potential raw material resources for several applications, like the production of building materials (concrete, geopolymers, bricks, and mortar). Valorization is a sustainable option for reprocessing, recycling, storing, and proactively managing mine waste towards the overall improvement of the environmental performance of the industry, all while yielding economic and social benefits within the context of the United Nations’ SDGs and the implementation of the Paris Agreement [10].

The most feasible alternatives for waste management are to reuse and recycle said waste [49]. The reuse of waste requires some quality consistency in comparison with the original state of the material. Reuse does not involve any biological, physical, or physicochemical transformations, thereby preserving natural resources while providing cheap products. Recycling, on the other hand, seeks to reintroduce waste, after undergoing alterations, to a specific production process as a raw material. Nonetheless, the most used practice in mining is treatment, disposal, and storage [15].

Usually, fine tailings from ore grinding are disposed of in tailing dams, an optimized, accessible, and environmentally safe disposal method that allows for future reprocessing when more advanced technologies are available. When geological and dam-facility stability, optimal land use, and operational safety are required, underground backfilling is an accepted alternative albeit an expensive one. Another solution is submarine tailing disposal, wherein tailings are placed in underwater marine bodies; this is the most controversial alternative, as while there is the favorable geochemical stabilization of sulfide mineral residues on the one hand, there are many operational risks on the other [15,50].

The global switch to innovative technologies and cleaner energy practices relies greatly on the exploration and extraction of several critical metals and minerals in ores. As a result, an intensification of mining activities is expected, requiring more land and producing more waste [16,39,40,41,42]. Taking into account that the mineral wastes from the mining industry alone already range in the order of billions of tons yearly [51], the immense increase in metal and mineral extraction over the last few decades [7] and the projected growth that will mirror the increase in population and consequent high consumption needs, an even greater production of corresponding mineral wastes is to be expected. Hence, the disposal and management of mine waste is of fundamental importance for addressing current and future environmental and social challenges [14].

A substantial part of mine waste is mine tailings, the fine-grained slurry consisting of milled and processed ore, processed water containing process chemicals, and their remains. The pervasive activity of ore extraction provides massive deposits/stockpiles of tailings, which is only anticipated to increase even further [17,23]. The quantity of tailings generated depends on the type of extracted ore, the chosen process for extraction, its composition, and the quality of the extracted ore, e.g., the extraction of low-quality copper ores can generate about 128 tons of tailings per ton of extracted copper, whereas high-quality ores can generate 1.5 tons of tailings for each ton of copper produced [7].

The European Commission has introduced the Best Available Technique (BAT) principles for minimizing the generation of tailing volumes and maximizing the opportunities for their alternative uses [24]. There are two ways of disposing of mine tailings: either by direct disposal into rivers, oceans, and lakes; or by indirect disposal in the form of slurry (25–30% solid) into a dam, cell, or confined reservoir [18]. Improper tailing disposal practices in the past have resulted in critical environmental hazards, such as the collapse of waste storage facilities [52,53,54], metal accumulation and soil contamination [55,56], acid mine drainage (AMD) [57], and dust mobilization [58]. Dam failures have been a major cause of environmental disasters and the loss of life in the past, with 237 worldwide reported incidents between 1917 and 2009 [18]. One such incident in China in 2008 caused the death of 277 people [59], while another in Brazil in 2019 led to the death of 206 more [45].

According to Cheng [60], ample utilization of tailings consists of four main aspects: reconcentration of tailings by recycling valuable metals or minerals in them; utilization as filler in backfilling mine sites; as a substitute for conventional building materials or as raw materials for production of building materials; and through the reclamation, rehabilitation, and regeneration of mined and tailing dams.

As a low-value material in slurry form, with a varying water content ranging from 18 to 32% [17,61,62], tailings can only present economic benefits if their large volumes are fully capitalized on. A promising viable approach is their use in building materials production, with a lot of research aiming in this direction as of late [60,63,64,65,66,67,68,69,70,71,72,73,74,75,76,77]. The rich silicon content of some types of tailings assists to that end, particularly in the case of producing cementing/cementitious materials [63,78]. However, as this function requires certain pozzolanic activity while tailings are typically low or not reactive at all, an activating pretreatment is therefore required, usually achieved by mechanical, chemical, thermal, or a combined activation process [60].

## 5. Mine Tailings in the Production of Concrete

In general, mining waste is geologically heterogeneous (sedimentary, metamorphic, and igneous rocks; soils; sludge; and loose sediments); highly variable in composition due to local geology and the extraction, mineral processing, and water treatment methods used; and mainly comprise crystalline phases [10,26]. They occupy vast areas of land and, if not suitably managed, evoke environmental and ecological disturbances and public health risks. The huge volumes of mineral waste produced, along with the exhaustion of natural resources from mining activities and their typical chemical composition (main components are SiO_2_, Al_2_O_3_, CaO, and Fe_2_O_3_) place mining waste as one of the most attractive candidates for use in construction materials, including concrete, backfilling, fired bricks, and ceramics, towards a sustainable and circular economy [44,72,73,79]. Regardless of their favorable chemical composition, mineral wastes are typically highly crystalline, a property that affects their ability to participate in chemical reactions within the cementitious matrix. Therefore, mining waste can have different functions as diverse concrete components, including sand, aggregates, and binders [10,14].

Tailings are the most prominent candidate as they emerge from milling already in the 1–500 µm range, whereas waste rock and sludge require additional comminution or dewatering before they can be assessed as binder constituents. Apart from having absorbed excavation and milling energy, the reuse of tailings contributes to the reduction in their volume, which requires long-term storage and is associated with dam-failure risks [17,52].

The research on recycling mine tailings for their use in concrete technology is currently gaining momentum due to the environmental benefits it could offer for the industry. However, the potential benefits from reducing clinker demand and diverting waste from storage are conditional on source-specific processing requirements, life-cycle burdens, and verification that contaminants remain acceptably controlled under relevant service-life and end-of-life conditions. Advancement of such research requires thorough analysis of mine tailings for their mineralogical, physical, and chemical properties and their environmental behavior (i.e., potential heavy metal leaching) [10]. Despite the notable amount of literature around reusing and recycling mine tailings as construction materials, the field remains limitedly covered, with important gaps that have yet to be tackled, predominantly those concerning optimization, efficiency, general guidelines, and technology transfer to a larger industrial scale [15]. This could well be linked to some of the main challenges and limitations of the concept. For example, the properties of mine tailings are highly variable, depending on the ore type and mining processes. Necessary specialized technologies are mandated by distinct physical and chemical characteristics to comply with the application requirements. The presence of significant concentrations of contaminants, such as heavy metals and sulfides, poses environmental and health risks, primarily through the weathering of minerals and leaching of trace elements. Economic considerations also remain decisive, as processing and treatments to render mine tailings suitable for building material usage often demand considerable investment, raising concerns regarding their cost-effectiveness, especially if considered alongside market acceptance and stakeholders’ reservations about performance, aesthetics, and perceived risks. Addressing these multifaceted challenges requires interdisciplinary collaboration among researchers, industry practitioners, policymakers, and community representatives, supported by robust regulatory frameworks and standards to guide the responsible and sustainable integration of mine tailings into concrete technology [10].

For the past few years, there has been flourishing interest in incorporating tailings in different building material productions, either as a raw material or as an additive or partial substitute to conventional materials. This includes the utilization of tailings in the production of alkali-activated binders [51,80], ceramics [31,81], backfilling paste [82,83], concrete, either as aggregate [64,72,84,85] or binder replacement [23,86,87], and cement-based mortars [67,88,89].

There is high variation regarding:

- The type of concrete studies focus on, e.g., traditional concrete [90,91,92,93], autoclaved aerated concrete (AAC) [64,75,94,95], and ultra-high-performance concrete (UHPC) [71,96].
- The method used for tailing reactivity enhancement, e.g., thermal, chemical, mechanical, and compound methods [7,17,22].
- The type of mine tailings investigated [61,97,98,99].
- The tailings’ properties linked to particular results.
- The concrete or cement properties and respective experimental procedures chosen to measure and benchmark the effects of tailing incorporation.

## 6. Mine Tailings as SCMs

The usage of mine tailings as SCMs has only recently gained research momentum, possibly due to the low initial reactivity of most tailings [17,23,61,87,89]. The great variability of mineralogical and chemical composition of tailings, along with the complexity of cement and concrete behaviors, might be the main roots of this scarcity. Hence, the necessity of such research is even more evident, and it will provide essential knowledge that will lead to more generalized guidelines and optimal paths for harnessing the potential of the enormous amounts of available tailings.

The suitability of mine tailings for applications as SCMs depends on their mineralogy and chemical composition, which vary a lot depending on the type of ore being extracted. For example, a chemical composition of bauxite [100], coal [101], graphite [102], gold [103], copper [104,105], iron [42,77], kaolinite [106], and lead–zinc tailings [88] is dominated by silicates, (with bauxite, coal, and kaolin tailings also containing a considerable amount of Al). Whereas phosphate [107], tungsten [61,108], and zinc tailings [109] contain significant amounts of Ca. Materials for SCMs are classified based on the modulus of hydraulicity [110], which should be 0.02–3.98, and the silica-to-alumina ratio of 0.86–9.12. Furthermore, according to EN 450-1 [111], pozzolans are required to have a minimum of 70% of summed SiO_2_, Al2O_3_, and Fe_2_O_3_, whereas EN 197-1 [112] mandates a minimum 50% summed reactive calcium oxide (CaO) and reactive silicon dioxide (SiO_2_). Nevertheless, these traditional indicators of hydraulicity and pozzolanicity are not sufficient to fully assess the suitability of tailings in partially replacing Portland cement due to their high crystallinity [67]. The aluminosilicates are not necessarily soluble and ready to participate in chemical reactions. Hence, mineralogical characterization is essential for initial evaluation of the reactivity and to decide about possible additional activation treatments [7,31]. For example, the aluminum bonds in phyllosilicates (e.g., kaolin, muscovite, phlogopite, etc.) are not as strong as the ones in tectosilicate feldspars [113,114].

Since there are no regulations or standards for the use of tailings as SCMs, for initial screenings, a comparison of chemical compositions, with limits set by standards for typically regulated SCMs (i.e., fly ash), can be used as a starting point. For instance, according to EN 450-1 [111], the organic content should be less than 5% (LOI550 < 5%), as a higher organic content may prolong the setting and hardening times of concrete due to high porosity resulting from its degradation [115]. Additionally, based on the same standard, SO_3_ and MgO should be kept below 3.5–4% and 4%, respectively, as they may affect volume stability due to expansion [30].

Physical properties, including morphology, specific surface area, and particle-size distribution, affect cement and concrete properties, i.e., water demand, setting time, hydration rate, and, consequently, strength development [116,117]. A well-graded grain size enables packing and improves the pore structure. There is variation in the particle sizes of tailings that ranges from sand to silt through to cement [7]. Finer fractions could lead to increased water demand and affect early strength formation [117,118]. Angular-shaped grains decrease workability and packing due to increased friction, but they can enhance bonding between the cement and aggregate interface [17]. Mine tailings can increase water demand not only by their high fineness and angular shape but also due to their high absorptivity [7]. In general, a higher specific surface area provides a larger available reaction area and therefore enhances reactivity [119,120].

Untreated mine tailings should not be treated as a single reactive class. Their function depends on their mineralogy, amorphous content, particle-size distribution, specific surface area, and morphology, as well as the cementitious environment. A screening study of 13 mine tailings found potential chemical contributions in five of them and potential physical contributions in three, while the remaining materials required pretreatments before their valid use as SCMs. Accordingly, this review classifies each study according to the function actually demonstrated (i.e., aggregate, filler, reactive SCM, or alkali-activated precursor). Activated tailings are described as reactive SCMs only where structural modification is accompanied by direct evidence of chemical reactivity [17,20,21,23,27,28,29,30,51].

Table 1 presents a functional and evidence-based classification by linking each use of mine tailings to its dominant mechanism, the evidence required for classification, and the representative studies. Aggregate replacement is distinguished from binder applications, filler-dominant materials are separated from tailings with demonstrated SCM reactivity, and alkali-activated precursors comprise a separate binder category instead of being interpreted as direct evidence of performance in Portland-cement blends [23,29,80,121].

When used as filler, tailings are reported to decrease mortar flow in an inversely proportional rate to the amount of incorporated tailings, with a constant water-to-binder ratio [124]. Zheng et al. [125] reported that when the mine tailings have a larger particle size than that of cement, the overall specific surface area abates and, subsequently, so does the water demand. The same study also reports a retarding effect on the hydrating mechanisms due to phosphorus ions, leading to a longer setting time. The results are not conclusive on whether the use of mine tailings as a filler improves mechanical properties. According to Siddique and Jang [124], adding 5%, 10%, 15%, and 20% molybdenum mine tailings increased compressive strength by 3%, 1%, 10%, and 16% and flexural strength by 2%, 5%, 24%, and 17%, respectively. An improvement in abrasion and impact resistance was reported as well when compared to the reference concrete mix. Additionally, all mixes had higher density, lower total porosity, and lower water absorption. The mixes with 10% tailings showed the best sulfate and acid attack resistance [124]. On the other hand, in the study conducted by Zheng et al. [125], when 30% of phosphate tailings were added, the measured compressive and flexural strengths were 35.9% and 25% lower. This was attributed to the dilution effect. Moreover, an increased drying shrinkage was observed when 10% of tailings were present [125]. The two studies cannot be ruled as contradictive; instead, they are evidence of the influence of mineralogy, mixture proportions, particle size, morphology, and hydration chemistry of the incorporated mine tailings in cement-based materials. It is important to remember that tailings may also behave as a reactive filler, so the reactivity factor should be considered [7].

Mine tailings have been studied as a cement replacement. Some reported a meshy or flocculent structure of the calcium–silicate–hydrate (C-S-H) in 90-day-old samples, in contrast with its fibrous structure in plain cement paste. There were also fewer portlandite crystals formed, and the Ca/Si ratio was lower [126,130]. On the contrary, the 3-day-old pastes showed a higher Ca/Si ratio due to the faster dissolution of Ca from Portland cement compared to the release of Si from tailings [126]. The ratio gradually reduces with age and hydration, leading to secondary reactions and the formation of the meshy C-S-H. A denser and more uniform overall microstructure was also reported. Replacing PC with 30% phosphate mine tailings caused increased bleeding and longer setting times, as well as a delayed hydration induction phase [125]. The retarding effect was related to the dissolution of phosphate. The thixotropy, plastic viscosity, and yield stress were significantly decreased with the increasing amount of phosphate tailings. A 10% replacement of PC with coal-mine tailings was shown to have no or only a marginal effect on the setting time [101]. Some early data indicate that there is a dependence of the induction period and hydration on the replacement of cement by tailings. It was suggested that, in the early stages of hydration, tailings contribute only as fillers [126]. The start and duration of the induction period, hydration, and rheological properties were observed to be highly affected by material fineness and reactivity. Generally, the presence of reactive tailings in cementitious matrices provides additional Si needed for later pozzolanic reaction and enables the formation of secondary C-S-H, but this can only occur at later ages, depending on the reactivity of the tailings [7].

Using mine tailings as a cement replacement inevitably affects the mechanical behavior of the end-product. It depends on the type of tailings, the replacement level, and the water-to-binder (w/b) ratio; the results from past studies on this are scattered. For example, Zheng et al. [125] replaced cement with 30% of phosphate tailings, which led to a reduction in compressive strength at 3, 28, and 90 days by 34.7%, 38.9%, and 40.7%, respectively. The flexural strength was lowered by 25%. A 60% replacement of PC with copper tailings reduced the flexural strength by 45% [126]. Others noticed a 41,5% compressive strength reduction when PC was replaced with 50% coal-mine tailings [132]. An optimum replacement level for copper tailings was observed to be 30%, and resulted in an increase in the compressive and split tensile strength, along with a higher elastic modulus by 5%, 12%, and 10%, respectively [89]. Replacement of PC with 15% of graphite tailings yielded a reduction in compressive strength of up to 40% for various w/b ratios [102]. There are a few studies that have investigated cement replacement by mine tailings in combination with other conventional SCMs and reported both significant increases and reductions in compressive strength [96,133,134]. Durability strongly depends on the microstructure, which is affected in different ways by different properties of mine tailings. Each durability index should be evaluated separately. Mechanochemically activated siliceous iron tailings used at 10–40% cement replacement improved impermeability relative to the control mixture, and 30% replacement improved frost resistance. In the same study, carbonation resistance was lower than the control at each replacement level and deteriorated as replacement increased [130]. Graphite-tailing mortar provides another exposure-specific result. Graphite tailings improved frost resistance, and a 20% addition gave the best combined mechanical and antifreeze response under the tested conditions [135]. This optimum cannot be transferred to another type of tailings, binder, or exposure without verification.

Existing studies have many points of variation, tailing function, replacement level, w/b ratio, fineness, activation treatment, curing conditions, testing age, control mixture, etc. Additionally, not all studies consistently document the same parameters; therefore, direct comparison is not always feasible. For instance, molybdenum-tailing mixes [124] showing a strength improvement and phosphate-tailing mixes [125] showing a strength reduction and hydration delay are neither contradictory, nor do they mark molybdenum tailings as a better solution to phosphate ones. The first explores the addition of tailing in mortars as a filler while the later investigates cement-based pastes. Different material functions, replacement levels, and measurements for the evaluation of tailing effects render the studies not directly comparably. Additionally, studies incorporating the same type of tailings might be difficult to directly compare. Liu et al. [126] and Esmaeili et al. [89] both investigated copper tailings, and both refer to high-energy milling for reactivity enhancement with replacement levels overlapping at some points. Yet the mill types and milling protocols differ, and neither study verifies the achieved reactivity increase or the resulting particle-size alteration; one investigates concrete mixes and the other pastes. As an example of the variations in previous studies, Table 2 consolidates the most indicative preceding evidence on mechanical and durability performance of various mine tailings, when used as a binder replacement, in Portland-cement systems, together with the activation routes, replacement levels, and reported reactivity. The table enables comparison across studies while keeping filler-dominant behavior, demonstrated chemical reactivity, and aggregate substitution analytically distinct.

From an environmental perspective, mine tailings can contain elevated concentrations of residual metals and metalloids because target metals commonly occur in sulfide-bearing ores. If improperly handled or disposed of, sulfide oxidation can generate acidic, metal-rich drainage, resulting in acid mine drainage (AMD) and contaminated effluents [15,17,57]. Heavy metals can show reduced mobility when tailings are incorporated into alkaline cement-based matrices [88,95,137,138] through precipitation, sorption, incorporation into hydration products, and physical encapsulation. However, this should be described as condition-dependent immobilization rather than irreversible fixation because release varies with contaminant speciation, binder chemistry, curing time, pore structure, and pore-water pH [137,139]. Carbonation can consume matrix alkalinity and alter metal speciation, either by decreasing or increasing its release, depending on the element and degree of carbonation [140,141,142,143]. Many tailings also contain sulfide minerals, including pyrite and pyrrhotite, whose oxidation may cause internal sulfate attack, swelling, cracking, loss of neutralization capacity, increased permeability, and, ultimately, greater metal release or AMD [15,17,26,50,57,144,145]. Although tailing mineral components may improve some mechanical or durability properties [22,146,147,148], these benefits should not be generalized; the relationships among mineralogical and chemical composition, mechanical properties, durability, and environmental performance should be evaluated for a variety of types of tailings and across several conditions. Additionally, transferability between different tailings, binders, climates and applications should be established.

## 7. Application of Mechanochemical Activation on Mine Tailings

Reactivity is strongly correlated with the structural stability of any material. Materials with a higher degree of microstructural defects, lattice distortion, or amorphous content are less stable and therefore more reactive [128]. Mineral waste, and mine tailings in particular, have a high crystallinity; therefore, they require some form of reactivity enhancement for use in cementitious systems as reactive components of the binder phase.

While the available research supports a focused discussion on MCA, it does not support an assumption that it is universally superior to other activation routes. The evidence base is uneven: alkali activation already has dedicated reviews and has been investigated across numerous silicate minerals, although many mine tailings remain insufficiently reactive under alkaline conditions and require pretreatment. Direct comparative studies also show that thermal activation can outperform mechanical treatment for certain coal and graphite tailings [31,33,145,149]. In contrast, MCA of mine tailings is an emerging research area in which only a limited number of systematic SCM studies have directly connected milling conditions, structural transformation, dissolution, and chemical reactivity [23].

Mechanochemical activation (MCA) is a treatment that is able to increase chemical reactivity through high-energy milling by inducing collisions, impacts, attrition, and compression of the material, resulting in amorphization, particle-size reduction, specific surface area increase, structural disorder in the crystal lattice, and chemical alterations [14,130,150,151,152]. High-energy grinding can produce two related albeit distinct effects. During comminution, the impact, compression, attrition, and shear of fracture particles can reduce particle sizes and increase the external surface area. These changes can improve packing or nucleation without proving chemical reaction. During MCA, changes related to the particle size of the material, provoked by the continued energy transferred from the milling medium to the material, can lead to changes, dislocations, bond rupture, phase transformation, defects, and amorphization that increase the chemical reactivity of the material [150,153,154,155]. Additionally, it is reported in the literature that due to high impact and friction forces developed between the material and the grinding media, localized high-temperature conditions at collision points may be observed, contributing to these chemical reactions [151].

The two effects require different evidence. Particle-size distribution and specific surface area characterize comminution, whereas quantitative X-ray diffraction along with complementary spectroscopic, thermal, or microscopic measurements can demonstrate structural disorder, dehydroxylation, or the loss of structural order. Peak attenuation or broadening should be described as evidence of reduced crystalline order rather than as proof of complete amorphization unless it is quantitatively evaluated and corroborated. Chemical consequences should be established separately through dissolution measurements, calcium-hydroxide consumption, calorimetry, or hydrate-phase evolution [23,121,156,157]. This distinction retains the original discussion of amorphization and defect generation while clarifying the evidence needed to demonstrate that these changes produced greater reactivity [121,151].

**Figure 3:**
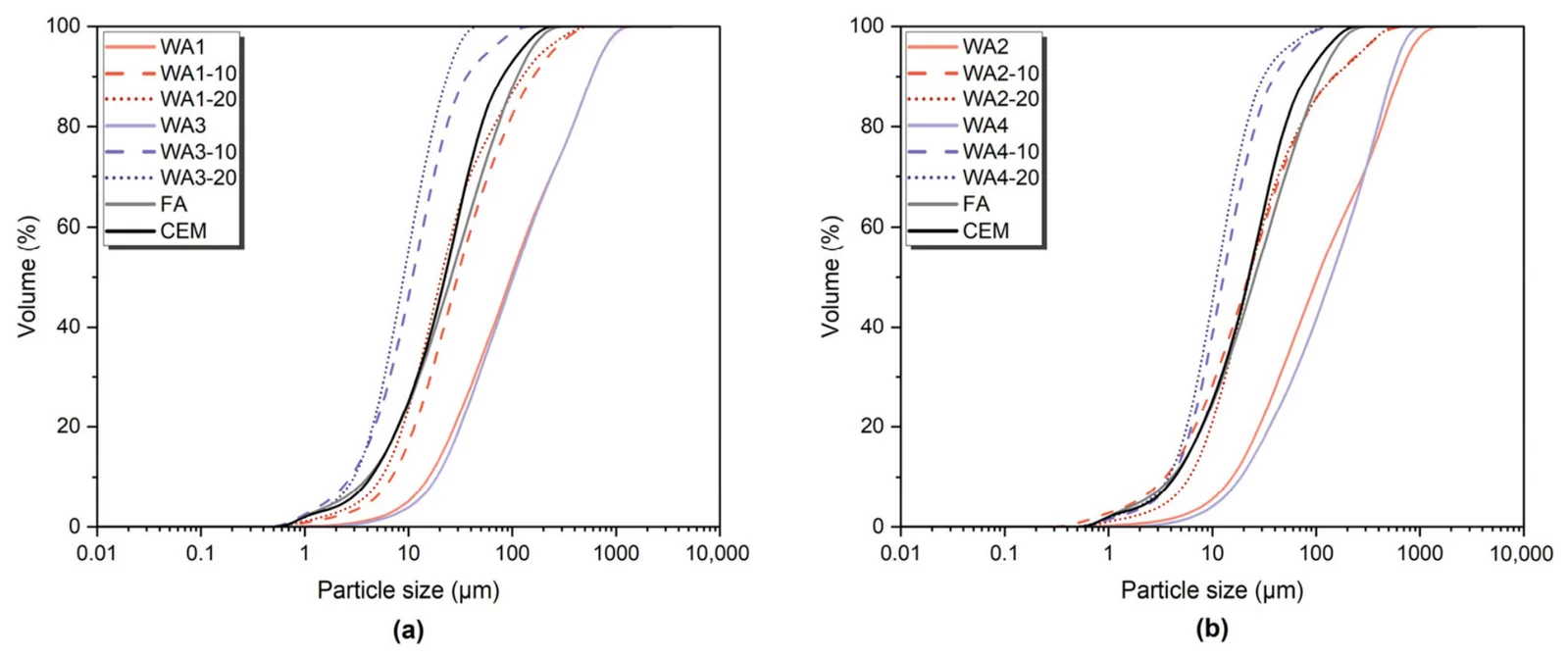
Cumulative particle-size distribution of four wood ashes—(**a**) WA1 and WA3 and (**b**) WA2 and WA4—after 10 and 20 min of high energy grinding compared to fly ash (FA) and Portland cement (CEM I). Reprinted from Ref. [131], published under a CC BY license.

**Figure 4:**
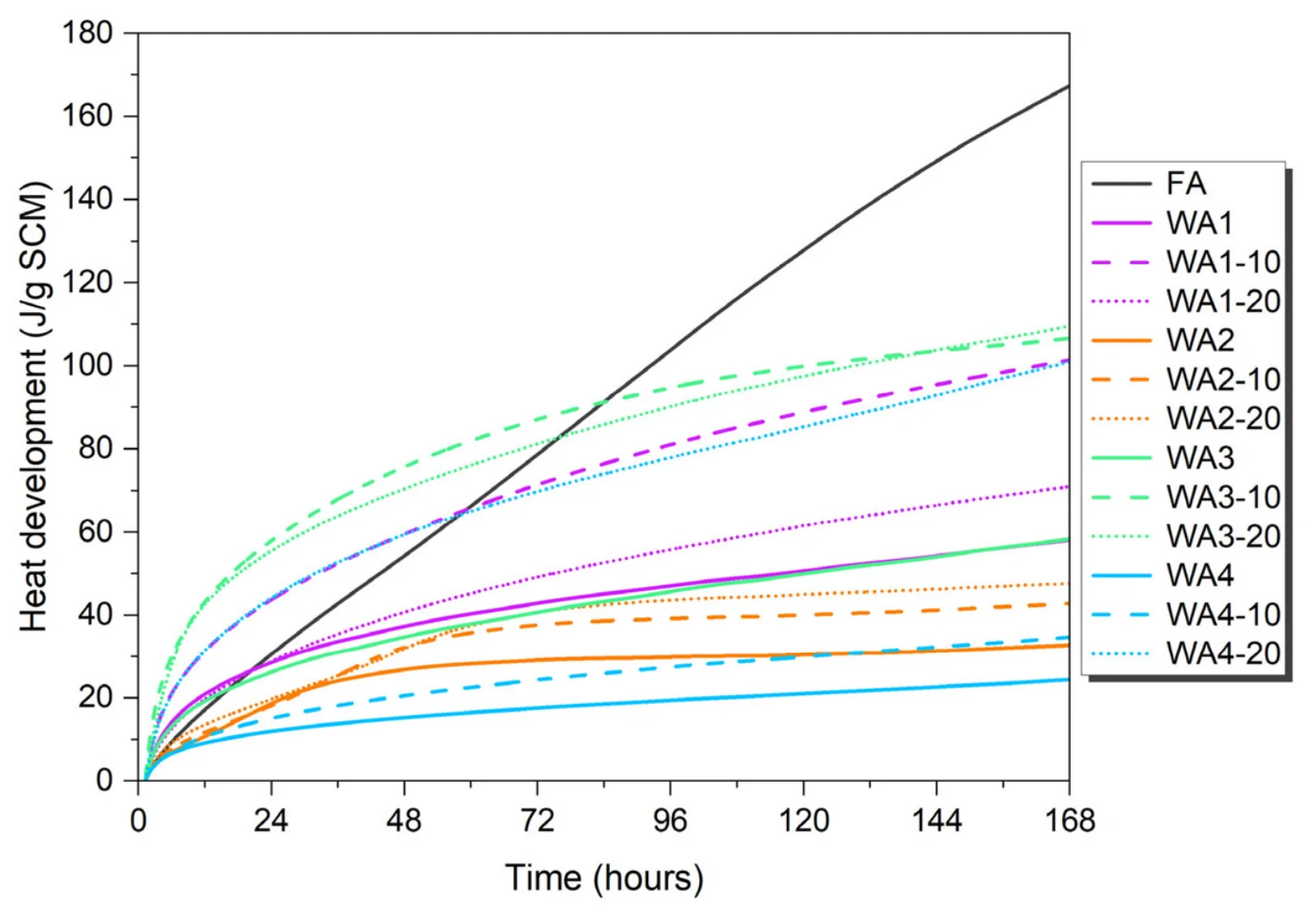
Seven days cumulative heat release of four wood ashes (WA1, WA2, WA3, WA4) after 10 and 20 min of high energy grinding compared to fly ash (FA). Reprinted from Ref. [131], published under a CC BY license.

Figure 3 [131] shows the effect of 10 and 20 min of high-energy grinding on the particle-size distribution of four wood ashes (WA1, WA2, WA3, WA4) in comparison with fly ash and cement. Figure 4 [131] presents the corresponding cumulative heat release according to the standardized R3 method [158,159]. In conjunction, they provide evidence of the two distinct effects of MCA mentioned above.

**Figure 5:**
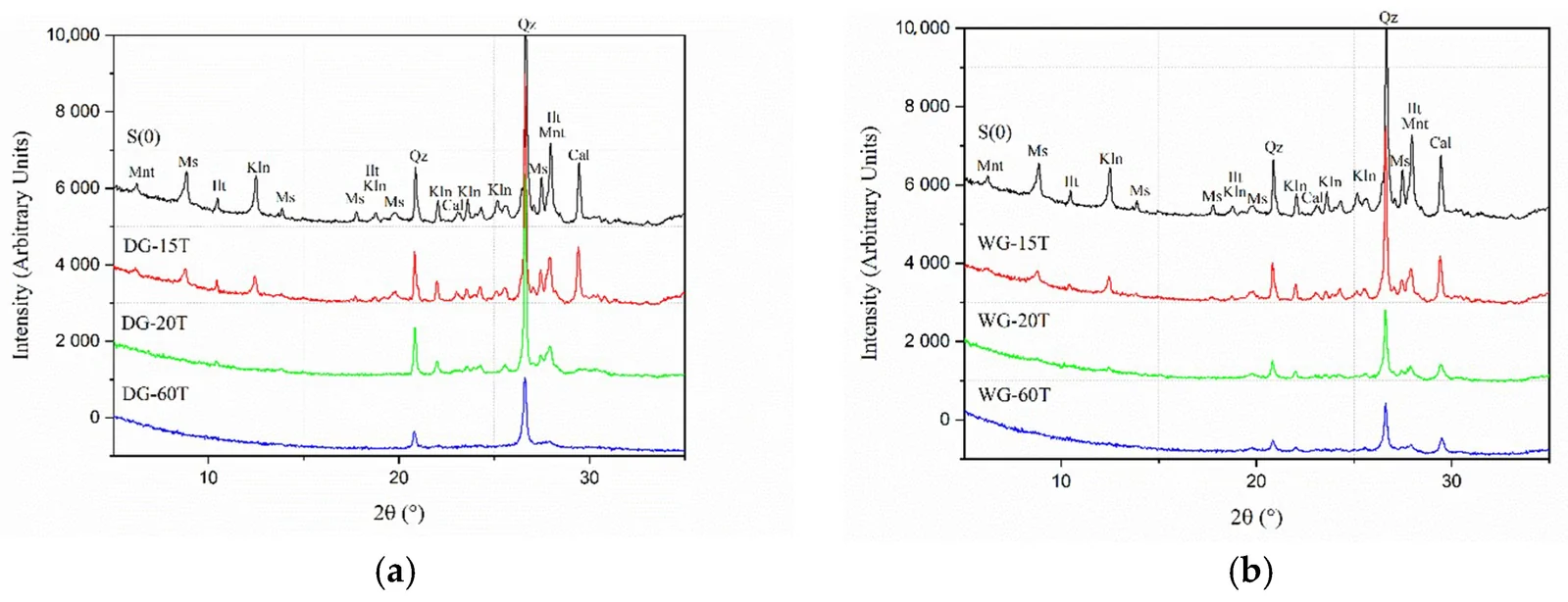
XRD patterns for different durations of grinding raw clay: (**a**) dry grinding for 15, 20, and 60 min; and (**b**) wet grinding for 15, 20, and 60 min. [Kln—kaolinite, Mnt—montmorillonite, Ms—muscovite, Ilt—illite, Qz—quartz, Cal—calcite.] Reprinted from Ref. [157], published under a CC BY license.

**Figure 6:**
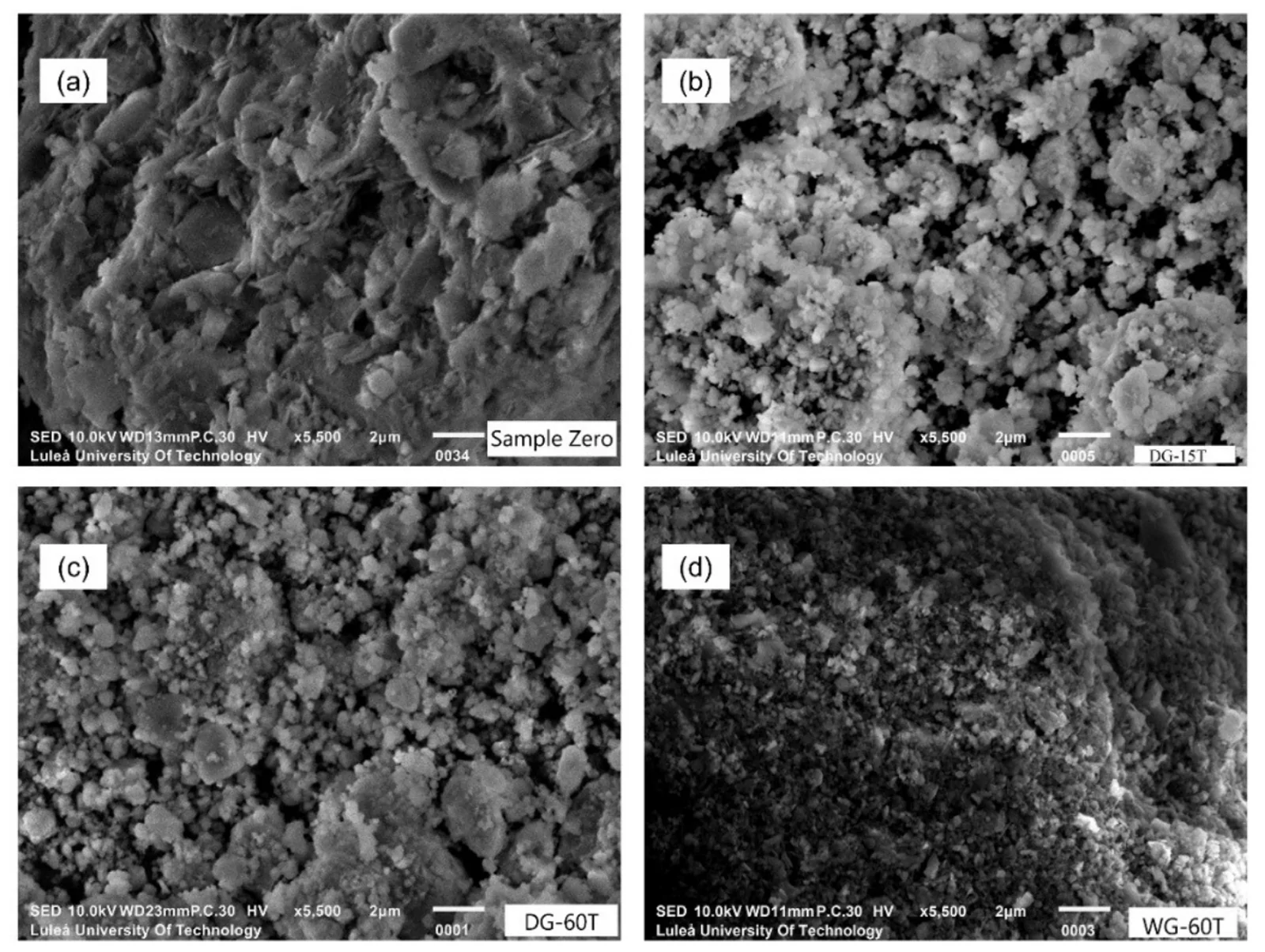
SEM-SE images of MCA clay: (**a**) raw material S0; (**b**) dry grinded for 15 min; (**c**) dry grinded for 60 min; (**d**) wet grinded for 60 min. Reprinted from Ref. [157], published under a CC BY license.

Another example of distinct evidence from different tests can be found in the study by Tole et al. [157], where XRD analysis was used to identify the changes in the crystallinity of the material (Figure 5) and Scanning Electron Microscopy (SEM) was used to observe the changes in the particles’ morphology (Figure 6) [157].

The above-mentioned examples not only indicate existing measurement methods for interpreting grinding-induced physical or structural changes due to MCA, but they also indicate the feasibility of applying MCA to different mineral waste and materials for the revalorization in cement blends, thus justifying the exploration of the same concept for mine tailings.

There is a variety of available equipment for high-energy grinding, i.e., ball mill, stirred ball mill, ring mill, nutating mill, planetary ball mill, vibratory ball mill, motor mill, jet mill, and discmill, with diverse energy inputs ranging from 13 to 165 kJ/g [160]. Each type imposes a different combination of compressive, shear, and friction stresses, providing diverse efficiency levels and a variety of choices depending on available input and the targeted output in terms of raw material hardness, amorphization, particle size, etc. [23,150,161,162,163,164,165]. Generally, planetary ball mills require shorter processing times, they are simple and safe to handle and they offer good reproducibility hence, they are the most common among laboratory applications [150,161,162].

MCA has been applied in many different research areas, i.e., materials engineering, pharmaceutics, preparative chemistry, agriculture, mineral processing, extractive metallurgy, and waste treatment [154,161,166,167,168]. Within construction material research, mechanochemical activation has been investigated for enhancing mineral wastes and by-products used as potential SCMs [23,60,92,103,128,129,130] and as precursors in geopolymer systems [80,121,152,164,169].

The reactivity of tailings has been reported to improve after high-energy grinding due to the creation of new surfaces, defects in the crystal lattice, and phase transformations [103,130,151,152]. Although the existing research is not extensive, there are several aspects that have been highlighted. Different minerals present in the source material behave differently under the same treatment. For example, after 80 min of grinding, the relative crystallinity of quartz reduced to 80%, whereas that of muscovite reduced to 37% [80,121,152,164,169]. Niu et al. [121] reported progressive structural degradation and increasing amorphous content in phlogopite-rich phosphate tailings as grinding proceeded, as based on XRD, TG/DTA, and mineral-liberation analyses. The structural change was accompanied by an increased dissolution of Si, Al, and K in an alkaline solution. These results support milling-induced degradation and enhanced reactivity. The response should consequently be interpreted phase by phase rather than through a single bulk activation value. Perumal et al. [80] associated the increase in amorphous content with changes in clinochlore, anorthite, and siderophyllite while also acknowledging uncertainty in quantitative XRD of complex multi-phase materials. In a mixed raw clay, Tole et al. [157] found that kaolinite, muscovite, and calcite were more readily disordered than quartz during dry grinding, whereas the quartz response was greater under wet-grinding conditions. Quartz, feldspars, phyllosilicates, and carbonates should therefore be tracked individually because they do not respond identically to a common milling treatment [23,121,157].

Researchers have documented the increase in the amorphous content of several mine tailings and minerals in relation to grinding time [7], while others have also used TEM to verify the amorphization of minerals pre- and post-treatment [128]. Apart from reducing particle size and increasing specific surface area, MCA, when applied in aluminosilicate compounds, can rupture O–H, Al–OH, Al–O–Si, and Si–O bonds, enhancing their reactivity in alkaline/cementitious environments [60,151,156]. High-energy milling can simultaneously increase fineness, lattice disorder, defect density, amorphous content, and aluminosilicate dissolution without relying exclusively on bulk calcination or chemical activators. MCA may be particularly useful for tailings containing some 2:1 clay minerals, such as micas or montmorillonite, which can be difficult to activate effectively through thermal treatment alone [23,170].

The effectiveness of MCA is nevertheless material- and equipment-specific. In the tailings study by Ramanathan et al. [23], ball-milled samples remained effectively inert under the tested conditions, whereas more intensive disk milling increased their reactivity, fineness, and amorphous contents. Reactivity was reported to approach a plateau after extended treatments. Prolonged milling can progress from fracture to agglomeration, or to caking, thereby limiting the gain in the accessible surface area and reducing process efficiency. A higher impact intensity can also increase the temperature and wear, while abrasion or corrosion of the grinding media can introduce contaminants into the material. Chromium contamination from stainless-steel equipment was observed during wet milling in one clay study [157].

Mechanochemical activation can avoid kiln-level bulk temperatures, but a low processing temperature does not by itself establish a low energy demand, low costs, or a low environmental impact. Energy values are comparable only when their system boundaries and functional outputs are equivalent. Modeled collision energy estimates energy delivered to the powder. Measured equipment electricity, and cradle-to-gate embodied energy are different quantities and should not be combined in a single ranking. Recent energy–reactivity analyses of planetary-milled clays found substantial variation in activation efficiency among milling conditions and diminishing reactivity gains at higher energy input, demonstrating that longer or more intensive milling does not necessarily provide a proportionate benefit [33]. Energy should be interpreted at a defined output, e.g., R^3^ reactivity, cement-replacement level, concrete-performance target, etc., because energy per ton alone does not establish useful activation [23,33,157]. Current laboratory evidence can guide the selection of scalable grinding conditions, but it does not yet establish universally favorable industrial energy demand or cost [33,157].

## 8. Conclusions

The above structured narrative review leads to the following principal conclusions:

1. Mine tailings cannot be treated as a uniform source of supplementary cementitious materials. Their function in cement-based systems, either as aggregates, fillers, reactive SCMs, or precursors for alkali-activated binders, depends on their source-specific mineralogy, chemical composition, amorphous content, particle-size distribution, morphology, and potentially hazardous constituents. Untreated tailings are frequently weakly reactive and consequently, each tailing stream requires case-specific characterization alongside performance- and risk-based qualification.
2. Selected mine tailings demonstrate laboratory-scale potential as partial replacements for Portland cement after appropriate characterization and, when necessary, activation. Reported effects on hydration, workability, strength, and durability vary with the tailing type, replacement level, mixture design, curing conditions, and test method. Additional systematic research is needed to establish mine tailings as generally feasible cement substitute and to verify their performance under industrial production and field exposure conditions.
3. Mechanochemical activation can improve the suitability of some tailings, but its effectiveness is material- and process-specific. Milling can reduce particle size and induce lattice disorder or partial amorphization, and these changes can increase dissolution or reactivity in some systems. However, more intensive or prolonged milling may produce diminishing reactivity gains, agglomeration, equipment wear, or contamination. Claims of chemical activation should therefore be supported by direct reactivity measurements rather than by fineness, X-ray diffraction peak broadening, or compressive strength alone.
4. Environmental and economic advantages have not yet been demonstrated on a general basis. Potential reductions in clinker use and tailing storage are yet to be validated and verified in future studies combining standardized reactivity and durability testing with pilot-scale processing, scale-up and wear assessment, leaching evaluation, comparative life-cycle assessment, and techno-economic analysis.

Generally, the available studies and data are not conclusive; instead, they are rather limited, scattered, and sparse. Apart from the different origin streams, there is great variability regarding mineralogical and chemical compositions, as well as several treatment methods for enhancing reactivity (one or more at a time), with numerous parameters that lead to different performances. It is evident that there is a the need for more than just proof-of-concept studies. An integrated investigation of processing techniques, replacement levels, technical performance, durability, energy demand, environmental risk, and industrial feasibility is clearly required.

Further and extensive research is needed to establish predictive correlations between tailings characteristics, mechanochemical activation conditions, and resulting concrete performance. It is important to have a more comprehensive understanding of how mineralogical composition, amorphous fraction, particle morphology, and defect generation influence hydration kinetics and macroscopic properties for a more realistic and scientifically grounded material design. There is a need for systematic comparative studies that isolate the effects of grinding duration, milling intensity, equipment type, and possible co-activation strategies on the reactivity of different-origin mine tailings. Similarly, long-term performance and durability should not be omitted. More evidence regarding chloride and sulfate resistance, carbonation, alkali silica reactions, drying shrinkage, creep, etc., will be essential for determining the type of end-application for these materials.

Overall, a variety of mine tailings have demonstrated, at a laboratory scale, potential as partial substitute for cement, particularly following mechanochemical activation, requiring further systematic investigation that addresses the multifactorial nature of this subject. Tailing sources, replacement levels, water-to-binder ratios, curing ages, control mixtures, testing procedures and outcomes differ substantially and are not reported consistently across studies. These preliminary and heterogeneous laboratory results remain case-specific and cannot lead to generalized conclusions or trends for mechanochemical activation and utilization of mine tailings in cement blends. However, they justify further investigation of the concept. They substantiate the importance of further targeted research that will clarify the intercorrelations between tailing mineralogy, chemical composition, mill type, grinding protocol, chemical reactivity, and cement-blend performance. This information is essential for determining their scalability, environmental benefits, and long-term field performance.

## Figures and Tables

**Table 1:** Functional classification of mine tailings’ uses in cementitious systems.

Material Function	Criteria	Required Evidence	Examples from Previous Research
Aggregate	Chemical reactivity is not required [122]	Replacement basis, granulometry, absorption correction, workability, strength, exposure-specific durability [122].	copper tailings [72] iron tailings [71,79,123]
Filler (untreated tailings in the binder fraction)	Filler/dilution/nucleation alterations unless chemical reaction is directly demonstrated [23,29]	Matched inert-filler control, particle-size distribution, specific surface area, calorimetry, calcium-hydroxide balance, dissolution, hydrate phases [23].	molybdenum tailings [124] phosphate tailings [125] copper tailing [126] 13 types of tailings [127]
Binder (yhermally activated)	Demonstrated phase transformation and pozzolanic response [92]	Activation temperature and duration, phase transformation, direct reactivity, strength, durability [92].	calcined oil sands [78] iron tailings [92] coal-mining waste [101,114]
Binder (mechanochemically activated)	Structural change linked to increased chemical reactivity [23]	Mill type, duration, speed, media, fineness, structural change, direct reactivity, mixture performance [23].	nickel/copper tailings [23] iron tailings [60,128,129] gold tailings [103] iron tailings [130]
Binder (alkali-activated)	Alkali-activated performance is not direct evidence of Portland-cement SCM performance [77,131]	Activator chemistry and proportions, precursor fraction, curing, strength, durability, separate testing in Portland cement before SCM classification [77].	copper/nickel, copper/zinc, iron tailings [51] phosphate tailings [107,121] silicate tailings [82]

**Table 2:** Indicative cases of mine tailings used as a binder replacement in cementitious systems, with key findings and limitations from selected studies.

Mine Tailings	Treatment	Replacement Level	Key Result	Durability	Comments
Ni–Cu & Quartz Tailings [23]	Ball mill (30 min, 450 rpm) Disk mill (1–15 min, 1500 rpm)	30% w/b 0.40	- Ball-milled samples remained inert - Disk milling increased fineness, amorphous content, dissolution, and R^3^ reactivity, with gains approaching a plateau after ~8 min	Not tested, identified as a gap	- Strong process–structure–reactivity evidence-only early-age paste validation
Iron-Ore Tailings [92]	Heat treatment 500 °C & 750 °C 40 min	10%, 20%, 30%	- Heat treatment transformed kaolinite toward metakaolinite - Pozzolanic tests were positive - Strength met requirements for several commercial cement classes	- Acid attack mass loss - Microstructural damage decreased as treatment temperature and tailing content increased	- Thermal transformation provides stronger SCM evidence than oxide composition alone
Gold Tailings [103]	Mechanical grinding (up to 80 min)	30%	- Muscovite relative crystallinity fell to 37% - 28 d activity index reached 78.99%, with C–S–H and ettringite formation	N/R *	- Direct structural and reaction evidence supports pozzolanic classification
13 Tailings [127]	No treatment	Various replacement levels	- Reduced workability by 3–19% - Strength generally fell as replacement increased - Five reached up to 10% higher 28 d strength at 20%	N/R	- Surface area and SiO_2_ were favorable, while larger sizes, high LOI, CaO, and CaCO_3_ were unfavorable but not fully predictive
Molybdenum Tailings [124]	No treatment	5%, 10%, 15%, 20%	- Compressive strength (+3%, +1%, +10%, +16%) - Flexural strength (+2%, +5%, +24%, +17%)	- Best sulfate and acid attack response at 10% replacement	- Favorable filler performance under one mix design - No proof of pozzolanicity
Iron Tailings [130]	MCA	10–40%	- Impermeability exceeded control mix at all levels - 30% replacement improved frost resistance	- Carbonation resistance was lower than the control and worsened as replacement increased	- Durability endpoints moved in opposite directions - Universal conclusion not possible
Copper Tailings [126]	Grinding	>30%	- Strength decreased as content and grinding time increased - Induction and pore structure changed	N/R	- Grinding did not automatically improved performance
Coal-Mining Waste [132]	Thermal activation	10–50%	- At 50%, chloride diffusion fell from 20 × 10^−12^ m^2^/s for OPC to 2.7 × 10^−12^ m^2^/s	- Strong chloride transport improvement	- Alumina-rich activated waste can improve chloride resistance despite high clinker replacement
Coal-Mining Waste [136]	Thermal activation	20%, 50%	- Tailings delayed chloride ingress and lowered the critical chloride content for corrosion initiation	- At 20%, mean reinforcement section loss was 21% lower than OPC	- Transport and corrosion-threshold effects must be reported
Graphite Tailings [135]	N/R	20% (optimal)	- Graphite tailings improved frost resistance - Selected 20–40% mixtures showed mechanical gains at specified freeze–thaw cycle counts	- Best combined mechanical and antifreeze response at 20%	- The optimum is exposure- and mixture-specific
Iron-Ore Tailings [128]	MCA	10%, 20%, 30%	- Grinding caused structural degradation - C–S–H and ettringite formation blends met the requirements for 32.5 composite Portland cement	N/R	- Supports activation for this source, but long-term durability remains a gap

* N/R: not reported.

## Data Availability

No new data were created or analyzed in this study. Data sharing is not applicable to this article.
